# High diversity of *Escherichia coli* causing invasive disease in neonates in Malawi poses challenges for O-antigen based vaccine approach

**DOI:** 10.1038/s43856-025-01007-1

**Published:** 2025-07-18

**Authors:** Oliver Pearse, Allan Zuza, Edith Tewesa, Patricia Siyabu, Alice J. Fraser, Jennifer Cornick, Kondwani Kawaza, Patrick Musicha, Nicholas R. Thomson, Nicholas A. Feasey, Eva Heinz

**Affiliations:** 1 Department of Clinical Sciences, Liverpool School of Tropical Medicine, Liverpool, UK; 2 Malawi-Liverpool-Wellcome Programme, Kamuzu University of Health Sciences, Blantyre, Malawi; 3 Queen Elizabeth Central Hospital, Blantyre, Malawi; 4 Department of Vector Biology, Liverpool School of Tropical Medicine, Liverpool, UK; 5 Institute of Infection, Veterinary and Ecological Sciences, University of Liverpool, Liverpool, UK; 6 Kamuzu University of Health Sciences, Blantyre, Malawi; 7 Parasites and Microbes Programme, Wellcome Sanger Institute, Hinxton, UK; 8 Department of Pathogen Molecular Biology, London School of Tropical Medicine and Hygiene, London, UK; 9 The School of Medicine, University of St. Andrews, St. Andrews, UK; 10 Strathclyde Institute for Pharmacy and Biomedical Science, University of Strathclyde, Glasgow, UK

**Keywords:** Bacterial infection, Conjugate vaccines, Infectious-disease epidemiology

## Abstract

**Background:**

*Escherichia coli* is an important cause of neonatal sepsis and the third most prevalent cause of neonatal infection in sub-Saharan Africa, often with negative outcomes. Development of maternally administered vaccines is under consideration, but to provide adequate protection, an understanding of serotypes causing invasive disease in this population is essential. We describe the genomic characteristics of a collection of neonatal *E. coli* isolates from a tertiary hospital in Blantyre, Malawi, with specific reference to potential protection by vaccines under development.

**Methods:**

Neonatal blood or cerebrospinal fluid cultures from 2012 to 2021 identified 208 *E. coli* isolates, and 169 could be recovered for sequencing.

**Results:**

Our data shows very high diversity in sequence types, LPS O-antigen-type and flagellar H-type, which all show temporal fluctuations and, as far as we are aware previously undescribed diversity, including ten putative novel O-types. Vaccines in clinical trials target the O-antigen but would only protect against less than half (37.9%) of neonatal sepsis cases in this population (EXPEC9V). An O-antigen-based vaccine would require 30 different O-types to protect against 80% of infections.

**Conclusions:**

Vaccines against neonatal sepsis in Africa are of considerable potential value, but their development requires larger studies to establish the diversity and stability over time of relevant O-types for this population.

## Plain language summary

Infection in newborn babies is a common cause of death in sub-Saharan Africa (SSA), a bacteria called *Escherichia coli* is one of the main causes. Vaccination of pregnant mothers may protect against *E. coli* infection. However, to provide adequate protection, an understanding of what types of *E. coli* cause infection in SSA is essential. We describe the features of *E. coli* causing infection in newborns in a hospital in Malawi and assess whether vaccines against components on the surface of these *E. coli* would protect against infection. We find that many different types of *E. coli* cause infection, meaning that vaccines in development would be ineffective as they don’t give protection against enough of the right *E. coli* types, and to develop effective vaccines may be complex. There is a need for larger such studies in SSA to enable a better understanding of the *E. coli* present and to enable effective vaccines to be developed.

## Abstract (web summary)

Pearse et al. use whole genome sequencing to describe the antigenic and overall diversity of 169 *Escherichia coli* isolates causing invasive infections in neonates in a single hospital in Malawi. High levels of O-antigen diversity are found, highlighting the difficulty of developing a vaccine for this patient population.

## Introduction

Neonatal infection is the third largest cause of neonatal death worldwide, currently at 18 per 1000 live births globally. Sustainable development goal 3.2^1^ aims to reduce this to 12 per 1000 live births by 2030. *Escherichia coli* is the third most prevalent cause of neonatal infection in sub-Saharan Africa^2^ and is a particularly important cause of early onset sepsis^3^ (EoS; sepsis before 72 h of life).

Antimicrobial resistant *E. coli* infection makes this worse and is an increasing problem in countries in sub-Saharan Africa^4^, such as Malawi. Limited access to antibiotics in the countries with the highest burden of infection makes these infections even more lethal^5^. In Malawi for example there are few antimicrobial choices for neonates beyond the first-line (benzylpenicillin and gentamicin) and second-line (ceftriaxone) therapies, for which there is already resistance amongst *E. coli* isolates^6^. In this context, innovative approaches to preventing infection with *E. coli* are required. One proposed approach is maternal administration of vaccines to give neonates passive immunity as is already used for *Bordetella pertussis*^7^ and being developed for Group B *Streptococcus*^8^. This would reduce the deaths and prolonged hospital stays of neonates linked to *E. coli* sepsis and meningitis, reduce the use of antimicrobials and thus the potential impact on population-level spread of antimicrobial resistance (AMR). However, the surface exposed structures that could be targeted by vaccination have high levels of diversity. There are currently 186 known O-polysaccharide antigens (O-types) for *E. coli*, 67 known capsular antigens (K-types) and 53 known flagellar antigens (H-types)^9^. O-types and H-types are most important as they are expressed by all *E. coli* and recognised by the immune system of the host, major K-types were explored initially as well^10–12^ but often poorly recognised by the immune system as they resemble host carbohydrate structures, and are likely to escape immune responses^13,14^. Crucially, only a small proportion of these serogroups are thought to cause the majority of invasive disease^15^ but this can vary depending on location and patient characteristics. A vaccine would therefore need to target the correct surface-exposed structures for the patient population in question. Importantly, H-type describes the protein-based diversity, which would thus need a different formulation to most vaccines against bacteria implemented or in trials, which mainly target polysaccharide structures (capsule or LPS O-antigens).

*E. coli* vaccines targeting the O-antigen are in development, including the Extra-intestinal Pathogenic *E. coli* 4-valent vaccine (ExPEC4V) (NCT02546960)^16^, and ExPEC10V (NCT03819049)^17,18^ which was reduced to ExPEC9V for stage III (NCT04899336)^19^, in clinical trials. These 4-valent and 10-valent (later 9-valent) vaccines have been produced to target the most prevalent serogroups causing invasive *E. coli* disease in older adults in high-income countries (HICs). Whether these candidates would reduce neonatal sepsis in sub-Saharan Africa is unknown, and the addition of other O-types would incur additional costs for clinical trials. In addition, different O-antigens are not necessarily equally easy to develop; one O-antigen already had to be removed from the initially 10-valent ExPEC10V, changing it to ExPEC9V, as the functional antibody assay for the O8 *E. coli* was not able to distinguish an immunological response to vaccination^18,19^.

There is little data on the antigenic diversity in *E. coli* causing invasive infection in neonates in sub-Saharan Africa, a population for which an understanding of this diversity is crucial if vaccination is to be feasible and to achieve equity in coverage across different regions. In this study, we present a genomic description of an unbiased collection of neonatal *E. coli* isolates, collected from 2012 to 2021 from neonates in a Malawian hospital, with a focus on O- and H-type diversity. We describe high levels of diversity and highlight that vaccines developed for high-income populations would offer poor protection in this population, emphasising the need for larger studies in this population.

## Methods

### Setting

Queen Elizabeth Central Hospital (QECH) is a government run tertiary referral hospital for the Southern Region of Malawi; care is free at the point of delivery. It directly serves urban Blantyre (population ~800,000 as of 2018 census data). Chatinkha nursery receives ~5000 admissions a year, with between 30 and 90 neonates on the ward at any one time. It admits neonates that have not gone home; either those that were born in QECH or those referred from another hospital. Paediatric nursery receives neonates that have been admitted from home via the paediatric Accident and Emergency (A&E). In both of these wards there is 24-h nursing care, and daily medical ward rounds. There is access to diagnostic blood and cerebrospinal fluid (CSF) culture, continuous positive airway pressure, oxygen, IV fluids, blood transfusion, radiology, and blood testing. There is also access to an on-site high-dependency unit (HDU). The paediatric intensive care unit (PICU/HDU) and paediatric surgical ward are located on the Mercy James Hospital, which opened in 2017. This is a surgical hospital on the QECH site but in a separate building, managed separately and philanthropically funded. At the surgical hospital there is additionally access to intensive care facilities (non-surgical patients from the main hospital may also be admitted to the intensive care), which allows for the use of vasopressors and intubation.

### Microbiological sampling and processing

Routine, quality-assured diagnostic blood culture services have been provided to the medical and paediatric wards by the Malawi-Liverpool-Wellcome Programme (MLW) since 1998. For details of sample processing, please see ‘Supplementary Methods’. Antimicrobial susceptibility testing was determined by the disc diffusion method (Oxoid, United Kingdom). All *E. coli* isolates were tested for their susceptibility to ampicillin, cefpodoxime (as a screen for bacteria with extended-spectrum beta-lactamase [ESBL] activity), chloramphenicol, ciprofloxacin, co-trimoxazole and gentamicin, and those that were resistant to cefpodoxime also had their sensitivity tested against amikacin, co-amoxiclav, cefoxitin, meropenem, pefloxacin and piperacillin-tazobactam. BSAC breakpoints were used until 2018, at which time EUCAST breakpoints were introduced. Details for the identification of other organisms are described elsewhere^4^. *E. coli* isolates were stored at −80 °C on microbank beads.

Between 1998 and 2010, diagnostic results were entered into ledgers that were later digitised, and from 2010, PreLink, a Laboratory Information Management system was used and information stored on an SQL database. The MLW database was screened from 2000 to 2021 to identify all cases of *E. coli* infection in the hospital, and all *E. coli* isolates (individual colony morphologies from a sample) from neonates (recorded as less than 29 days old or as 1 month old on ledgers) in the period from September 2012 to March 2021 were selected for whole genome sequencing (WGS). This time period was chosen as this was the time period for which we had consistent metadata at the time of WGS. Metadata used in the study was extracted from this database.

### Whole genome sequencing

A single microbank bead was removed from all selected *E. coli* isolates, which was thawed, streaked on MacConkey’s media and this media incubated for 18–24 h at 37 °C. Plates with growth of a single colony type were prepared for short-read WGS at 364 plex on the Novaseq SP generating 150 bp paired-end reads. For details of DNA preparation, please see ‘Supplementary Methods’.

Long-read sequencing was performed on a MinION MK1B sequencing device (ONT, U.K.). Library preparation was carried out according to the manufacturers protocol, using the ligation sequencing kit (SQK-LSK109) and Native Barcoding Expansion Kits (EXP-NBD104; all ONT). Sequencing was carried out using a FLOW-MIN106 R9.4.1 flow cell (ONT). Two samples did not produce sufficient data and were re-sequenced using the Native Barcoding Kit 24 V14 (SQK-NBD114.24, ONT), following the manufacturer’s instructions. Sequencing was then carried out using a R10.4.1 Flongle flow cell (ONT).

### Genomic sequence analyses

Sequence QC and assembly details are outlined in the Supplementary Methods. Sequence type (ST) was determined using mlst v2.23^20,21^ (https://github.com/tseemann/mlst). If no ST could be assigned, samples were submitted to EnteroBase^22^ (v1.2.0) to assign a novel ST. AMRFinderPlus v3.10.40 was used to identify AMR genes^23,24^. SRST2 v0.2.0^25^ with the EcOH database^26^ was used to determine the O- and H-types for the bacterial isolates. Where an isolate had more than one predicted O- or H-type, both sets were counted. We further aimed to confirm the subtypes (O1A, O6A, O18A, O25B) of relevance for the vaccine, which are not distinguished from their related (but immunologically non-identical) subtypes when using automated prediction via SRST2. For the distinction of O1A and O1B, sequences were compared to the specific primers and probe used previously^27^. For O18, there are four distinct subtypes described: O18A, O18A1, O18B and O18B1. We distinguished O18ac (~O18A/A1) from O18ab (~O18B/B1) using the presence/absence of an IS element inserted at a location immediately upstream of the *wzz* gene^28^ and as we were unable to distinguish O18A and O18A1 further we refer to it as O18A?. To distinguish O25A and O25B, we assessed the operon structure as described previously to distinguish these types^29^. We were not able to identify any description on the genetic differences of O6A and other O6 sub-types (referred to hereafter as O6?).

To further investigate their antigenic structure we performed long-read sequencing using the Oxford Nanopore platform for 14 selected isolates for which we had failed to identify their O-type. The O:H loci were investigated initially using the ECTyper^30^ which uses assemblies as input, which yielded comparable results to the srst2 EcOH search (see Supplementary Data 3); we then assessed these isolates further manually to determine the exact structure of these untyped O-Ag types.

### Statistics and reproducibility

Statistical analysis was done in the R statistical programming language^31^, using Rstudio^32^ and the packages here^33^, tidyverse^34^ and lubridate^35^. Graphical data representation was performed using the packages ggplot2^36^, ghibli^37^, RColorBrewer^38^, pals^39^, MetBrewer^40^, gggenes^41^ and compound figures created by ggpubr^42^. Missing data was excluded.

*E. coli* sample positivity was calculated as the number of samples that were positive for *E. coli* per 1000 samples taken (blood culture or CSF) according to equation 1 below.

$${P}={n}\div{N}\times 1000$$

Where *P* is the sample positivity rate, *n* is the number of *E. coli* infections and *N* is the total number of blood culture or CSF samples taken.

Comparisons of categorical data were performed using the Chi-squared test in R, with simulated *p* values (based on 2000 Monte Carlo simulations) if the expected frequency of any category was less than five.

The change in AMR over time was estimated by regressing resistance pattern of isolates against year of occurrence using a generalised linear model in R (glm function) with the binomial family and a logit link statement. Isolates were given a binary categorisation of 1 if they were resistant or had intermediate resistance to an antibiotic and 0 if they were sensitive. The regression coefficient for year, standard error of this coefficient and *p* values are presented. Plots of AMR trends over time had lines of best fit that utilised a linear model.

For the rarefaction curves in the main text, lines showing hypothetical coverage for vaccines based on the *n* most frequent O-types or H-types were compared to lines showing the hypothetical coverage based on the EXPEC4V and EXPEC9V vaccine O-types; Supplementary Fig. 3 furthermore shows our analysis for the original ExPEC10V composition. For isolates with more than one allele for H-type, both were included in the rarefaction curve. For H-types the rarefaction curve goes above 1 as there were multiple isolates with more than one H-type. For O-types there was only one O-type per isolate (there was one O-type with both O8 and O160 sections, but it is unclear whether this expresses both sugar molecules or is a hybrid O-antigen) so the rarefaction curve only goes to 1. The analysis was repeated (Supplementary Fig. 4) but only counting each isolate with multiple H-types once (the H-type that had the highest population frequency was chosen). The other allele was ignored, as theoretical protection from the vaccine was assumed by the H-type or O-type that was most prevalent.

### Ethics statement

This study was ethically approved by the Kamuzu University of Health Sciences College of Medicine Research Ethics Committee (COMREC P.06.20.3071). As this is a retrospective study of samples and data collected during clinical care the need for informed consent was waived by COMREC. The ID numbers used in this manuscript are specimen IDs generated by the MLW diagnostic laboratory and not patient IDs; only the research team and members of the clinical staff in the hospital who have access to the password-protected laboratory information management system at MLW and would be able to make the link to an individual.

### Ethics and inclusivity statement

With the exception of AJF, NRT, and EH, all authors are or were until recently based full-time in Blantyre, Malawi, where they work at either or both Queen Elizabeth Central Hospital (QECH) or the Kamuzu University of Health Sciences and its affiliate the Malawi Liverpool Wellcome Program (MLW). The study was conceived by the team of clinical researchers who were working in or with the neonatal unit of QECH, and addresses a major concern for public health specifically in sub-Saharan Africa and in particular in Malawi; neonatal sepsis caused by multidrug-resistant Gram-negative bacteria. Our team includes clinical and microbiology scientists from the Kamuzu University of Health Sciences and its affiliate the Malawi Liverpool Wellcome Program (MLW) as well as two clinical care staff working at QECH. Our team also includes Malawian junior researchers and junior team leaders, contributing to their career progression and continuity for research in Malawi. Materials were transferred under the Nagoya Protocol agreed between the Malawi Ministry of Health and the Wellcome Sanger Institute. We address a research question of urgent relevance for the local population; given considerations of the introduction of a maternally administered vaccine against *E. coli* neonatal sepsis, we highlight the urgent need to include truly global data to ensure appropriate vaccine targets are included for the target population.

### Reporting summary

Further information on research design is available in the Nature Portfolio Reporting Summary linked to this article.

## Results

### Clinical characteristics

**Fig. 1:**
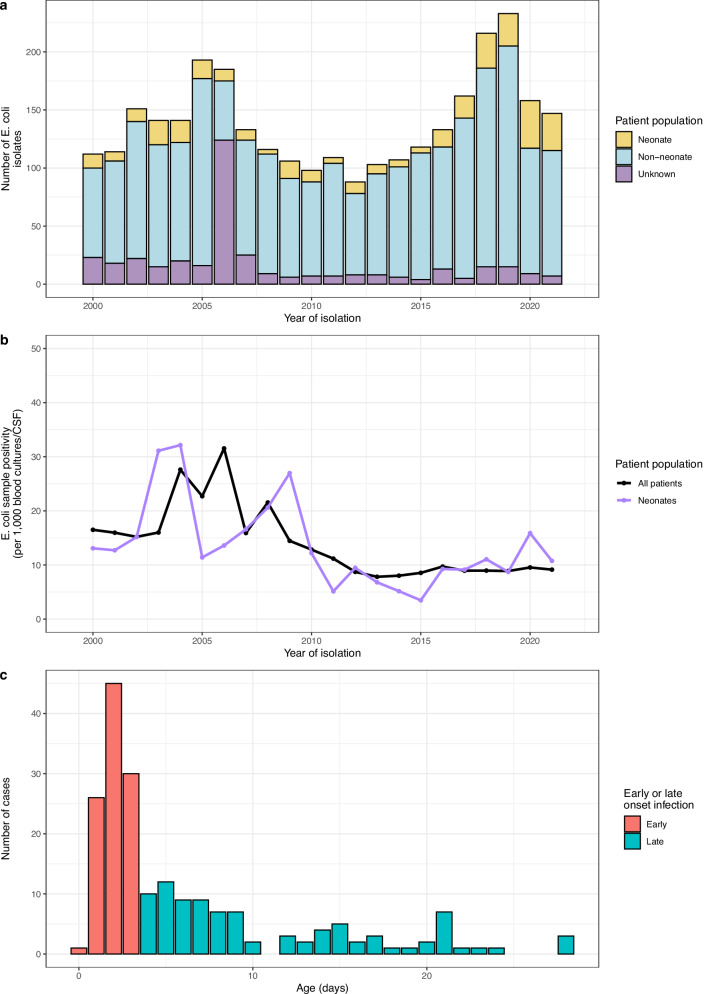
A summary of the E. coli cases by year and age of patient. **a** Numbers of *E. coli* cases per year at QECH. Bars represent the crude frequency of *E. coli* infection for each year from 2000 to 2021, with the different colours representing the different age groups of the patients. **b** Blood culture and CSF positivity rate (per 1000 blood culture or CSF samples) of *E. coli* in neonates and the entire patient population (including neonates). **c** Age range of neonates in the current study, colours highlighting early (<72 h of life) or late (>72 h of life) onset infection.

There were 3394 *E. coli* isolated from 264,692 blood culture and CSF tests over the period from 2000 to 2021. The number of cases of *E. coli* per year for all ages ranged from 88 to 233, for neonates ranged from 4 to 41 (Fig. 1a). The number of blood culture or CSF samples taken per year for all ages ranged from 2796 to 26230 with an average of 11029 in a year. The number of blood culture or CSF samples taken per year for neonates ranged from 77 to 3211 with an average of 1228 per year. The positivity rate per 1000 blood culture or CSF samples for all age groups was highest in 2006 and lowest in 2013, with an average positivity rate of 14.1/1000 samples/year. For neonates, it was highest in 2004 and lowest in 2015, with a similar average positivity rate of 13.7/1000 samples (Fig. 1b).

**Fig. 2:**
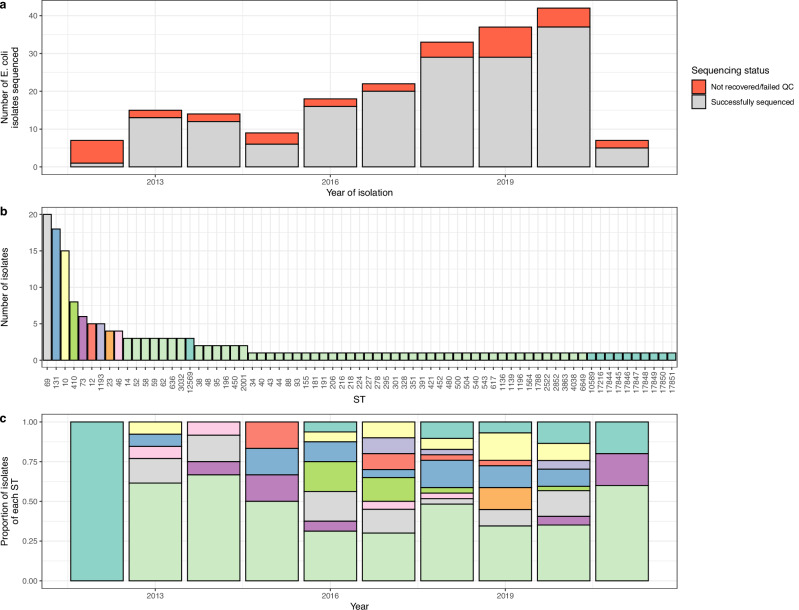
Quality control (QC) and sequence types (STs) of study isolates. **a** Number of *E. coli* isolates selected for WGS for this current study. 2012 and 2021 were years for which isolates were only selected from part of the year. **b** Frequency of different STs in the collection, most frequently isolated STs are colour coded, previously untyped STs highlighted in teal. **c** Frequency of the different STs by year, colour code as in (**b**).

We identified 207 *E. coli* isolated from 203 individual samples (four had two separate *E. coli* morphologies identified) in the period from September 2012 to March 2021 (Fig. 2c); 95/200 (47.5%) were female, with a median age of 3 [IQR 2–8] days (Fig. 1c). EOS accounted for 111/203 (54.7%) of cases, with late onset sepsis accounting for the rest (Fig. 1c). Of these isolates 163/201 (81.1%) were cultured from blood and 38/201 (18.9%) from CSF. There were 110/200 (55%) cases from the Chatinkha nursery, 62/200 (31%) from paediatric nursery with the rest coming from other wards (Supplementary Fig. 1). Of these 207 isolates, 169 could be regrown and passed all QC steps, and were included in our analyses (Fig. 2a).

### Population structure

There were 71 different STs represented in the collection (Fig. 2a, b; 60 previously type and 11 previously untyped, though these have now been assigned an ST). The most frequently isolated STs were ST69 with 20/169 (11.8%) isolates, ST131 with 18/169 (10.7%) isolates, ST10 with 15/169 (8.9%) isolates and ST410 with 8/169 (4.7%) isolates. There were 13/169 (7.7%) isolates from 11 STs which were previously unreported using MLST (Fig. 2b, c). Over half of the observed STs were only represented by a single isolate (38/71, 53.5%) showing that neonates tested here were exposed to and infected by a highly diverse pool of *E. coli* that span the species phylogeny. ST410 was disproportionately found in the CSF rather than blood culture samples (6/8 [75%]), compared to ST69 (2/20 [10%]), ST131 (2/18 [11%]) and ST10 (2/14 [14%]) which were found primarily in blood culture samples.

Importantly, the ST diversity was also highly variable over time. 50/71 (70.4%) STs were only found in a single year, and 7/71 (9.9%) STs were found in only two years, with each year showing a similar pattern of high diversity during the entire study period. Frequently occurring STs were also prominent in different years (e.g. ST410 in 2016 and 2017), only ST69 was consistently isolated and was the most frequently isolated or joint most frequently isolated ST in 5 out of 7 of the years with more than ten isolates. Even the most prevalent STs like ST69 or ST131 however fluctuated over time, with numbers between 1/29 (3%) and 6/35 (17%) for ST69, and 1/20 (5%) and 5/29 (17%) for ST131, respectively, with no clear trend over time observable for any of the main STs. We also note that previously unreported STs are derived from a range of years, including the most recent data. This indicates that these are not representing older lineages that might not be covered well in databases consisting mainly of recent samples, but indicating a high undescribed diversity circulating at the present time. Four samples were polymicrobial. They both contained two different colony morphologies and two different STs. Two of these samples contained ST10 along with another ST and one contained ST69 along with another ST.

### O-antigen and H-antigen diversity

**Fig. 3:**
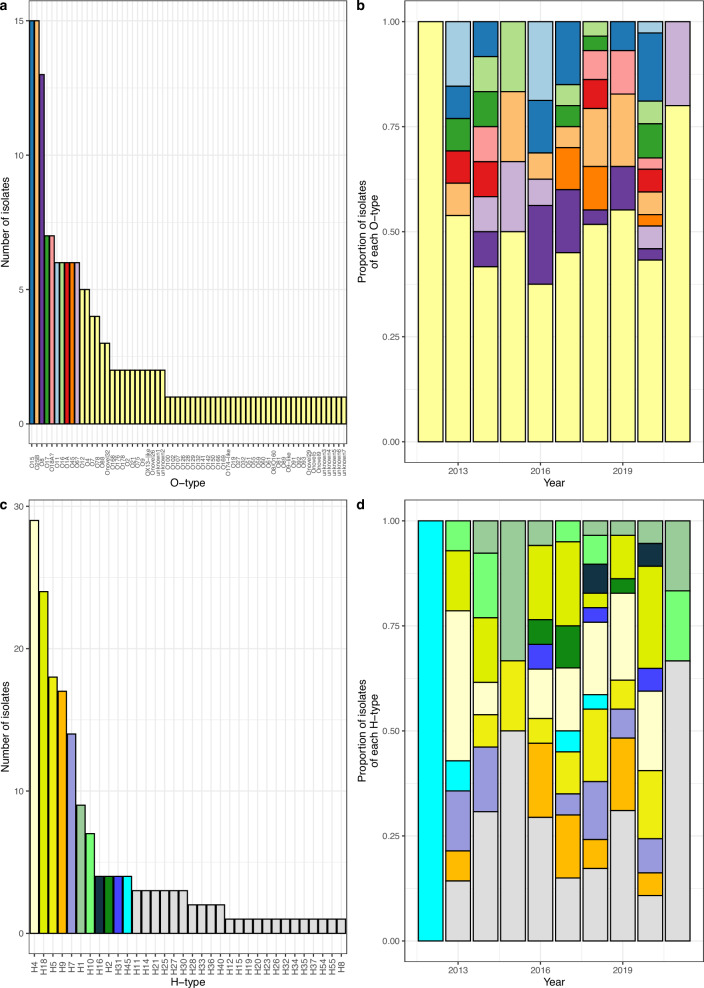
O-type and H-type diversity. **a** A bar chart showing the frequency of the different O-types. **b** A bar chart showing the proportion of isolates per year that had different O-types. The colours are the same as those represented in (**a**). **c** A bar chart showing the frequency of the different H-types. Where an isolate had more than one H-type gene, this was counted twice. **d** A bar chart showing the proportion of isolates per year that had different H-types. The colours are the same as those represented in (**c**).

There were 63 O-types found in the collection, none of which were identified in more than 10% of the isolates. The most frequently isolated were O15 with 15/169 (8.9%) isolates, O25B with 15/169 (8.9%) isolates and O8 with 13/169 (7.7%) isolates (Fig. 3a). These same O-types (O15, O25B and O8) were also the only ones found in greater than 75% of the years (6 out of 7 or more) that had more than 10 isolates (Fig. 3b). Like ST there was no sign of the population becoming increasingly dominated by any O-type over time, the composition between years differed strongly (Fig. 2b). There were no years in which any O-type represented greater than 20% of the isolates, the largest proportion of isolates belonging to a single O-type per year were O11 and O8 which were both associated with 3/16 (18.8%) of all cases in 2016 (Fig. 3b).

We performed long-read sequencing for 14 isolates which either had no O-type call or had multiple O-type calls, to determine the genomic region between *galF* and *gnd* where the O-antigen type locus is usually found in *E. coli* (Supplementary Fig. 2). Having no O-antigen is highly unusual and leads to increased susceptibility to antimicrobial stress, and thus seems unlikely to be present in clinical isolates. Ten different O-antigen loci were revealed in these 14 isolates, with four of them represented by two isolates each. Two isolates were confirmed as O178 and one appeared to be a hybrid of O8 and O160, whilst seven of these ten O-types were so far undescribed (Supplementary Fig. 2) and one showed similarity to the OX-13 gene in *Salmonella* (BKRHXR).

There were 34 H-types found in the collection, of which 4 H-types were each identified in more than 10% of isolates. These were H4 with 29/173 (16.8%), H18 with 24/173 (13.9%), H5 with 18/173 (10.4%) isolates and H9 with 17/173 (9.8%) isolates (Fig. 3c). Five H-types were found in at least 75% of the years (6 out of 7 or more) that had more than 10 isolates (H4, H18, H5, H9 and H7). H4 and H18 were the only H-types responsible for greater than 20% of the isolates in any year with more than 10 isolates (in two years each) but no H-type was identified in over 50% of isolates in a single year. The largest proportion of isolates belonging to a single H-type in a single year was H4 with 5/14 (35.7%) of cases in 2013 (Fig. 3d). There were 4/169 (2.4%) isolates that had more than one H-type and may be able to undergo phase variation for immune escape, hence the denominator of 173 above. ST410 isolates with O8 and H9 were more likely to be found in CSF (6/33 [18.2%]) than bloodstream (2/133 [1.5%]; *χ*^2^ = 16.03, *p* = 0.0015).

Excluding STs for which there was just one isolate, we examined whether multiple O-types or H-types were found in isolates of a single ST. The median number of O-types per ST for the STs that met this criterion was 2, with 10/23 (43%) encoding for just a single O-type and 13/23 (57%) encoding for multiple O-types. The median number of H-types per ST for the STs that met our criteria was 1 with 14/26 (54%) encoding for just a single H-type and 12/26 (46%) encoding for multiple H-types. Three of the most frequently occurring STs encoded for multiple O-types and H-types. ST10 showed the highest diversity of O-types and H-types, with 10 different O-types and 7 different H-types. ST131 covered 3 different O-types (O25B, O11, and O16) and 2 different H-types (H4 and H5), which occurred from 2013 to 2020 and often with multiple O-types or H-types in the same year. ST69 isolates included 4 different O-types and 2 different H-types. ST410 on the other hand occurred frequently from 2016 to 2020 but all isolates were encoded for only a single O-type (08) and a single H-type (H9).

### Predicted vaccine coverage

**Fig. 4:**
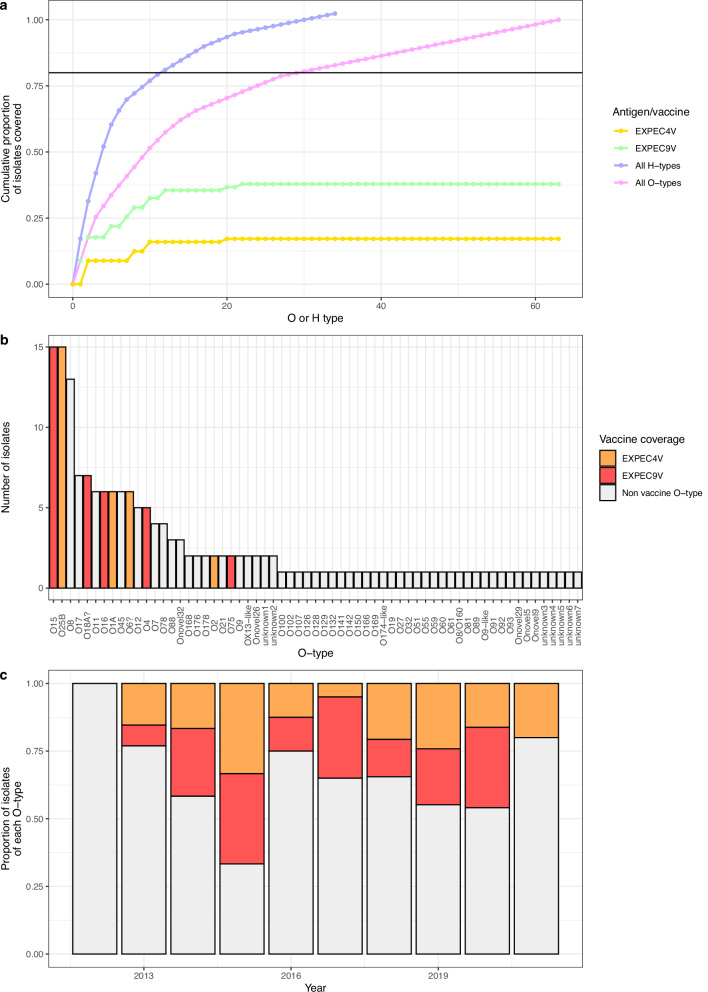
Theoretical vaccine coverage for extra-intestinal pathogenic *E. coli* 9-valent (EXPEC9V) and EXPEC4V. **a** Rarefaction curve showing the theoretical protection given against vaccines covering the most frequently isolated H-types and O-types, as well as the potential protection given by the EXPEC9V and EXPEC4V. The horizontal line shows the point at which 80% of isolates would be covered. For isolates with more than one H-type both were counted, there were multiple isolates with more than one H-type so the line for H-type goes above 1. Supplementary Fig. 3 shows the same graph but where isolates had more than one H-type called they were only counted once. **b** A bar chart showing the frequency of the different O-types with the O-types covered by EXPEC4V and the additional coverage on top of this offered by EXPEC9V highlighted. **c** A bar chart showing the proportion of isolates per year that had different O-types. The colours are the same as those represented in (**b**).

The EXPEC9V conjugate vaccine (which covers the O1A, O2, O4, O6A, O15, O16, O18A, O25B, and O75) might be expected to confer immunity to up to 64/169 (37.9%) of these cases, the original 10 V composition (including O8) would have covered up to 77/169 (45.6%), demonstrating a loss of 7.7% by the removal of just one O-antigen of high prevalence in our setting (Fig. 4a and Supplementary Fig. 3). The EXPEC4V vaccination (which covers O1A, O2, O6A, and O25B) would cover have covered 29/169 (17.2%) of cases (assuming the O6 isolates are of the O6A subtype, something that we have not been able to confirm; Fig. 4a, b; ‘Methods’). Analyzing the data by year (including only years with 10 isolates or greater) the EXPEC9V vaccine covered fewer than 50% of the isolates’ O-types in every year, and 2 out of 7 years covered less than 30% of the vaccine O-types, with the lowest coverage in 2013 where only 23% of the isolates were from vaccine O-types, and importantly we observe no major change in coverage over time (Fig. 4c). The EXPEC4V vaccine covered less than 30% of the isolates’ O-types in every year, and 5 out of 7 years covered less than 20% of the vaccine O-types, with the lowest coverage in 2017 where only 5% of the isolates were from vaccine O-types, with fluctuations over time that do not indicate any improvement in coverage in future (Fig. 4c). Regarding the isolates which were resistant to first- and second line antimicrobial therapy (benzylpenicillin, gentamicin and ceftriaxone), the EXPEC9V vaccine would be expected to confer immunity to 13/34 (38.2%) of these cases and the EXPEC4V vaccine would be expected to confer immunity to 9/34 (26.5%) of these isolates.

To cover 80% of cases, an O-antigen based vaccine would need to offer protection against the top 30 O-types. In contrast, to cover the top 80% of these cases, a H-type vaccine would need to cover the top 12 H-types (Fig. 4a) if a protein-based vaccine were considered. If the four most frequently occurring O-types in our setting were selected from our cohort for a vaccine (O15, O25B, O8, and O17) this vaccine would cover 50/169 (29.6%) of cases, ranging from 21 to 40% per year, and the nine most frequently occurring O-types (O15, O25B, O8, O17, O18A?, O11, O16, O1A and O45) would represent just under half of the isolates 81/169 (47.9%), ranging from 33 to 67% per year, with numbers fluctuating over our study period showing no indication that there would be an increase in coverage in future.

### Antimicrobial resistance and plasmid replicons

At the time of the study the first line treatment for neonatal sepsis and meningitis in QECH was benzylpenicillin and gentamicin, with second line treatment ceftriaxone. *E. coli* isolates with resistance to all three antibiotics were therefore difficult to treat (42/194 [21.6%]). There was occasional but limited use of amikacin or meropenem for neonates with proven or high suspicion of ceftriaxone resistance or who were very unwell. The use of meropenem and amikacin increased over the study period. Isolates were frequently multi-drug resistant (MDR; resistant to antimicrobials in four or more antimicrobial categories; 60/200 [30%]) and extensively-drug resistant (XDR; resistant to antimicrobials in all but two or fewer antimicrobial categories tested; 29/200 [14.5%]).

We identified AMR genes against all major classes of antibiotics and several efflux pump systems, in line with the phenotypic resistances detected (Supplementary Fig. 5; Supplementary Data 5). The number of AMR genes varied by ST, with ST410 (mean 24.0, SD 1.9) and ST131 (mean 20.6, SD 4.8) having the greatest number of average AMR genes per isolate. ST10 had a lower number of AMR genes per isolate (mean 8.9, SD 1.6), whilst ST69 was intermediate (mean 12.9, SD 1.5). ST410 was present only from 2016 onwards which may partly explain the higher number of resistance genes, whilst the other STs, including ST131 were present throughout the study period.

**Fig. 5:**
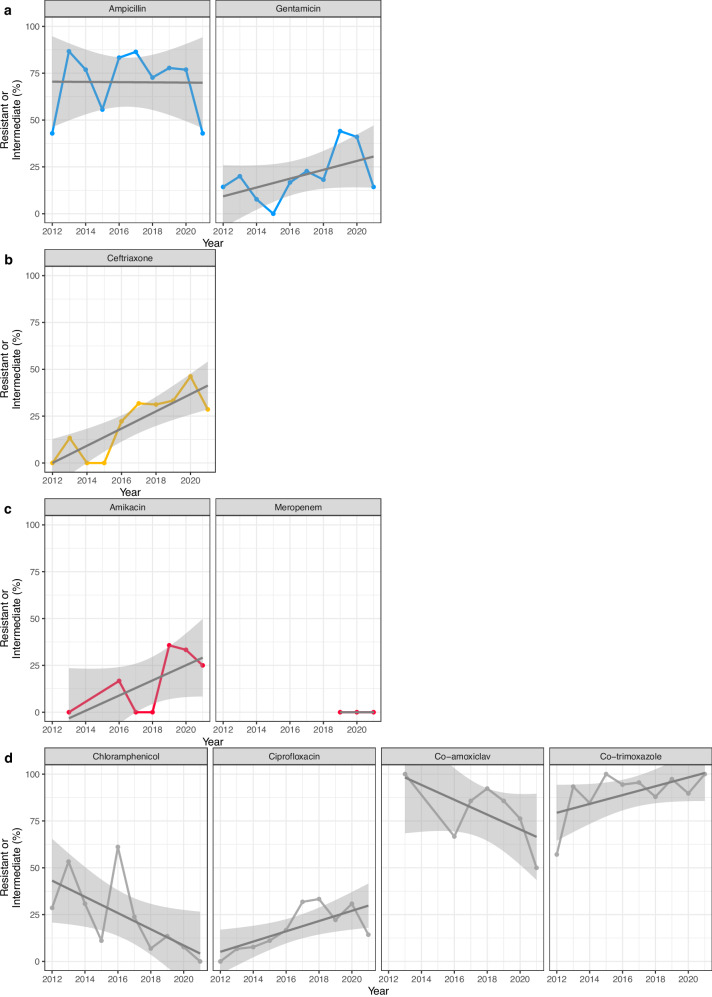
The proportion of *E. coli* isolates that were phenotypically resistant to different antibiotics by year. **a** First-line antibiotics for neonatal infection (*n* = 190 for ampicillin, *n* = 197 for gentamicin). **b** Second-line antibiotics for neonatal infection (*n* = 198). **c** Occasionally used antibiotics for neonatal infection (*n* = 67 for amikacin, *n* = 39 for meropenem). **d** Antibiotics not used in neonates, but used elsewhere in the hospital or in the community (*n* = 195 for chloramphenicol, *n* = 199 for ciprofloxacin, *n* = 200 for co-amoxiclav, *n* = 200 for co-trimoxazole). Trend lines represent linear regression (solid line) with 95% confidence intervals (shaded area).

The number of *E. coli* isolates that were resistant to ampicillin was 143/190 (75.3%) and was stable over the period (−0.3% change per year; S.E = 6.7%; *p* = 0.96; Fig. 5a). *bla*_EC_ genes, which are chromosomally encoded in *E. coli*, were found in all 169 isolates (*bla*_EC-15_, *bla*_EC-5_, *bla*_EC-18_ and *bla*_EC-8_). The most frequently occurring plasmid-encoded beta-lactamase penicillinase genes included *bla*_TEM-1_ found in 121/169 (71.6%) isolates, and *bla*_OXA-1_ found in 21/169 (12.4%) of isolates (Supplementary Fig. 4). Whilst only 49/197 (24.9%) were resistant to gentamicin, this however showed a temporal trend, increasing from 3/15 (20%) in 2013 to 16/39 (41%) in 2020 (22.8% change per year; S.E. = 7.8%; *p* = 0.0035; Fig. 5a). Gentamicin resistance mechanisms were mainly variants of the gene *aac*(3), *aac*(3)-IId found in 23/169 (13.6%) isolates and the *aac*(3)-IIe gene found in 12/169 (7.1%) isolates (Supplementary Fig. 5).

Ceftriaxone was the second-line treatment at the time of study, and 55/198 (27.8%) were resistant to ceftriaxone. This increased over the period from 0/7 (0%) in 2012 to 18/39 (46.2%) in 2020 (31.8% change per year; S.E. = 8.3%; *p* = 0.00014; Fig. 5b); and 42/55 (76.4%) of the isolates resistant to ceftriaxone were also resistant to ampicillin and gentamicin, hampering the effectiveness of all first- and second-line treatments. The increase in ceftriaxone resistance was due to the widespread ESBL gene *bla*_CTX-M-15_, which was detected in 39/169 (23.0%) isolates (Supplementary Fig. 5), whilst other alleles, *bla*_CTX-M-14_ and *bla*_CTX-M-27_, could be identified in only 1/169 (0.6%) isolate each, both from 2018 (Supplementary Fig. 3). ESBL genes were frequent in ST410 (8/8 [100%]) and ST131 (9/18 [50%]) as observed in other studies^43,44^, and infrequent in ST69 (3/20 [15%]) and ST10 isolates (1/15 [7%]). The proportion of isolates from late onset infection resistant to ceftriaxone was 16.9% (95% CI 3.5–30.2%) higher than from early onset cases (37.2% vs 20.4%, *χ*^2^ = 6.2; *p* = 0.012); the same pattern was observed for gentamicin with 15.8% (95% CI 2.8–28.7%) more resistant isolates in late onset infections compared to early onset cases (33.6% vs 17.9%, *χ*^2^ = 5.8; *p* = 0.016), whereas similar proportions were observed for ampicillin with 78.5% vs 73.2% in late and early, respectively (*χ*^2^ = 0.5; *p* = 0.50).

Alternatives for isolates resistant against all of the above antimicrobials are amikacin or carbapenems, and so far only a small proportion, 7/67 (10.4%), were resistant to amikacin. This increased from 1/6 (17%) in 2016 (amikacin was not routinely tested until 2016) to 7/21 (33%) in 2019 (52.7% change per year; S.E. = 25.9%; *p* = 0.042; Fig. 5c). The main gene identified was the amikacin resistance gene *aac*(6’)-Ib-cr5 in 21/170 (12.4%) isolates (Supplementary Fig. 5). Of the 42 isolates resistant to all first and second-line agents 11/42 (26.2%) were causing meningitis and a further 5/42 (11.9%) were resistant to amikacin, leaving only meropenem as effective treatment for these (amikacin does not reliably penetrate the blood-brain barrier). In line with the low levels of carbapenem resistance identified in other studies from this setting, no isolates showed phenotypic meropenem resistance.

Other antimicrobials are not regularly used on the neonatal unit but are tested for routinely for *E. coli*. Fluoroquinolones are still widely used in other wards, and 45/199 (22.6%) were resistant to ciprofloxacin which increased over the period from 0/7 (0%) in 2012 to 12/39 (30.8%) in 2020 (20.0% change per year; S.E. = 8.0%; *p* = 0.013; Fig. 5d). Chloramphenicol resistance has been observed in other isolates in this setting to decrease, and in line with this we observed 41/195 (21%) isolates resistant to chloramphenicol with a decreasing trend, from 2/7 (28.6%) in 2012 to 3/39 (7.7%) in 2020 (−30.5% change per year; *p* = 0.000027; Fig. 5d). Overall 54/67 (80.6%) were resistant to co-amoxiclav (not used on the neonatal unit and infrequently used in other hospital wards) and the proportion decreased slightly over the period (−20.7% change per year; S.E. = 21.1%; *p* = 0.33; Fig. 5d). Resistance to co-trimoxazole (used as prophylaxis against Pneumocystis pneumonia in HIV patients) was high over the period at 183/200 (91.5%) and increased slightly (14.9% change per year; S.E. = 9.8%; *p* = 0.13; Supplementary Fig. 5). Details on resistance mechanisms against these are provided in the Supplementary Materials.

There were 34 different plasmid replicons found in the dataset. The most frequently identified were the commonly detected IncF plasmid replicons that frequently carry resistance cassettes, with IncFIB_AP001918 found in 107/169 (63.3%) of isolates, IncFI found in 91/169 (53.8%) of isolates and IncFII_p found in 46/169 (27.2%) of isolates. Multiple different plasmid replicons were also found of the Col, IncH, and IncX types, with other types found less frequently.

## Discussion

This study highlights the challenges of controlling neonatal sepsis with vaccines in a low income setting when there is a paucity of data from this region describing the nature and diversity of isolates causing disease. We present the trends in predicted serotype epidemiology of a collection of *E. coli* isolated from neonates in a single, large teaching hospital in Malawi from 2012 to 2021. Our study reveals that O-antigen vaccines would need a high valency (30 O-types) to achieve protection against greater than 80% of isolates, and vaccines in current development for use in elderly populations in high-income countries would offer protection against less than half of the *E. coli* isolated in this study.

Our study, consistent with similar studies from high-income countries^45,46^, found approximately 50% of *E. coli* cases to be from early-onset sepsis (EoS) and 50% from late-onset sepsis (LoS). We found higher rates of ceftriaxone and gentamicin resistance in the LoS cases compared to the EoS cases (though this finding is not typical of other studies^47,48^), which might imply that in our setting these two groups have different epidemiology (e.g. EoS cases being maternally transmitted and LoS cases deriving from the hospital environment or from infection in the community). Almost a fifth of our isolates were from neonatal meningitis cases, and *E. coli* is an important cause of meningitis in neonates, including in low-income countries^49^. Neonatal meningitis is particularly concerning as it is associated with greater morbidity and mortality, requires longer treatment (minimum 21 days of antimicrobial therapy for *E. coli* meningitis compared to 7 days for bloodstream infection) and certain drugs such as amikacin cannot be used for meningitis due to concerns with poor blood-brain barrier penetration, leaving very few options for isolates resistant to third-generation cephalosporins.

The numbers of *E. coli* cases per 1000 blood culture or CSF tests in all age groups decreased from the period 2000 to 2012 but was relatively stable from 2012 onwards (which is the time period for which we had genomic data). There were peaks in the absolute numbers of cases in 2005 and 2019, although the peak in 2019 appears to be primarily related to greater patient numbers as there was no increase in the number of cases per 1000 blood culture or CSF tests done. This contrasts with *K. pneumoniae* at the same site over the same period^50^ which showed a large peak in numbers in 2019.

We saw very high ST diversity, with many STs occurring only once and eleven STs that were not present in the MLST database. This high ST diversity which was consistent over the whole study period may indicate that neonates are exposed to diverse sources of *E. coli*, reflecting the circulation of *E. coli* between the environment, humans and animals in our setting which differs markedly from One Health studies in high-income countries^51,52^; driven by limited access to water, sanitation and hygiene (WASH) and the much closer living conditions between humans and household-associated animals^53–55^. It also illustrates the need for more studies of *E. coli* diversity from sub-Saharan Africa, where the patient pool differs markedly from HICs where studies often focus on neonatal intensive care units (NICUs) which are rare in low-income countriess, and pre-term births which form a large proportion of cases in HICs have very low survival rates in low-income settings^56–58^. Although the STs with the highest numbers in our study are part of globally prevalent high-risk clones^59^ (ST131, ST10, ST69 and ST410), no ST was persistently present as a major lineage in our study. Our findings are comparable to previous findings from the same site in Malawi over a longer period^60^ and another study from Lilongwe^61^ which also found these STs to be common, and similar high diversity (e.g. 37 STs from 57 *E. coli* isolates in the BARNARDS study that analysed neonatal cases in four African and three Southeast Asian countries)^59^. ST410 has been associated with increased mortality in the Malawian context as one study found that all 8/8 (100%) of patients in the study that had bloodstream infection with ST410 died^43^ vs 135/326 (41%) of the overall cohort. In our study ST410 was associated with meningitis, the ESBL genes *bla*_CTX-M-15_ and fluoroquinolone resistance genes, leaving only meropenem as a treatment option.

The vaccines against *E. coli* which are in clinical trial stages target O-antigens. Whilst being developed to protect against invasive *E. coli* disease (NCT04899336, NCT02546960) in adults with a history of UTI in high-income settings, these or similar O-antigen-based vaccines could feasibly be administered to mothers to prevent neonatal sepsis. The choice of O-antigen glyco-conjugate vaccines is based on the knowledge that O-antigens are the major cell surface component of *E. coli*^62^ and appear to be essential for *E. coli* survival in human serum^63^. There are also multiple other glyco-conjugate vaccines that have been successful (including *Haemophilus influenzae* type b^64^, *Streptococcus pneumoniae*^65^ and *Neisseria meningitidis*^66^, though these are all based on bacterial capsule rather than O-antigen). Our study indicated high O-type and H-type diversity, with large flux of both, and no O-type or H-type representing the majority of cases in any year. As is the case in other collections^26^ there was higher diversity of O-type than H-type, meaning vaccine approaches targeting O-type require a much higher valency than those targeting H-types to protect against a similar proportion of isolates (30 O-types vs 12 H-types to protect against 80% of isolates). There were also 10 previously undescribed O-types and no undescribed H-types in our cohort. This higher diversity (including some that are previously undescribed) may call into question the practicality of using an O-type-based vaccine approach in our patient population and may direct efforts towards exploring vaccines based on other antigens. It is important to note however that H-type (flagella) is a protein-based target and would thus require a different strategy for vaccine production, as well as assessment for whether a protective antibody response can be raised using this target.

The EXPEC9V vaccine was designed for an elderly population in HICs, and for this role, it is likely to be effective. It was initially designed as a 10-valent vaccine; however, the serotype O8 was removed after the functional antibody assay did not work^19^. In our cohort, this change would have had a large effect on the utility of such a vaccine (dropping the proportion of isolates protected against from 45.6 to 37.9%). O8 was the third most frequently occurring O-type in our cohort, it was enriched amongst our meningitis cases and was present in all our ST410 isolates (which were highly AMR) and thus seems to be particularly common in high-consequence infections. Its removal would therefore drastically reduce the likely impact of this vaccine in our setting. Other studies in the target population for this vaccine (elderly adults in high-income countries) have shown good coverage against invasive cases (64.7^67^–67.5%^68^). The O-types in this vaccine appear wholly appropriate for this patient population. Interestingly, one of these studies^67^, though it was conducted across three continents and seven countries reported lower O-type diversity than in our collection (49 O-types; 47 identified O-types as well as two unknown O-types compared to the 63 distinct O-types found in our collection). This might reflect differences in the target setting as well as the patient population. Most appropriate to compare to our study is a study on a cohort of paediatric (most cases were from neonates) *E. coli* meningitis cases from France, where O1, O18, O45 and O7 were the most common O-types^69^ Amongst these, O18 was the only O-type found frequently in our study whilst other O-types such as O17, O12 and O11 were frequently found in our study but not in that one.

We also identified nine O-types and one combined O-type that were not identified by the EcOH database or ECTyper, highlighting our incomplete overview of O-antigen diversity, and that we lack knowledge of how frequently new types emerge. The high number of potentially uncharacterised O-types furthermore emphasises the need to perform serotyping and WGS on more isolates from sub-Saharan Africa as there might be substantial undescribed diversity. One of the unknown O-types was similar to the OX-13 antigen on *Salmonella enterica*. There are some O-types that are known to be shared by both *S. enterica* and *E. coli* (*E. coli* O-types O55, O111 and O157^70,71^). This is likely another O-type that is shared by both bacterial species. One O-type appeared to have sections from both the O8 and O160 O-type sugar molecules, it is not clear whether this is a hybrid of the two sugar molecules or whether this organism would be able to express both molecules.

Phenotypic AMR increased for several different antibiotics over the study period (ceftriaxone, co-trimoxazole, gentamicin, ciprofloxacin and amikacin) and was explained by a number of different genomic mechanisms. Ceftriaxone had been introduced in QECH as standard therapy for many infections in 2004, so this introduction alone cannot be responsible for the increase in resistance seen here. This is a global trend and whilst rates of AMR for *E. coli* are lower than for *Klebsiella pneumoniae* at the same site^4,50^, they are of significant concern. We identified no isolates with carbapenem resistance, which is not surprising as these genes are not widespread in Malawi, however, a previous study has identified a single carbapenemase carrying *E. coli*^72^. Chloramphenicol and co-amoxiclav resistance decreased over the study period. Chloramphenicol however is contraindicated in neonates and thus does not provide a treatment alternative. The increase in AMR, particularly in a setting where watch and reserve antibiotics are often unavailable due to cost, highlights the importance of prevention of neonatal infection in the first place with strategies such as vaccines or investment in improvements in infection, prevention and control (IPC).

### Strengths and limitations

A limitation of this study is the relatively low numbers and the fact that it was performed at a single site in a tertiary referral centre. It is therefore likely that it is not completely representative of the *E. coli* population causing invasive disease in neonates in Blantyre, Malawi; and not representative of sub-Saharan Africa. We also had limited demographics and metadata, which precluded detailed analysis or description of the patient population. However it represents a rare collection of *E. coli* genomes from neonates in sub-Saharan Africa, highlights important differences to HIC serotype distributions, and illustrates the fact that more investment and research is required to fully understand the diversity of *E. coli* causing neonatal infection in this setting.

In conclusion, the ongoing burden of neonatal sepsis combined with worsening AMR in *E. coli* motivates the development of *E. coli* vaccines for this population. However, the prevalent O-types in this collection from sub-Saharan Africa are highly diverse, potentially uncharacterised, and different to those currently covered by vaccines in clinical trials. If maternally administered vaccines are to be developed, they need to be based on robust genomic surveillance of prevalent antigens and temporal trends in this population, and far more data from sub-Saharan Africa is required to ensure equity of coverage compared to vaccines developed for HICs. Development of a suitable vaccine will be a lengthy process, and until a successful product is available other methods of preventing mortality from neonatal sepsis such as IPC and early recognition of neonatal infection should be urgently supported.

## Supplementary information

Supplementary information

Description of Additional Supplementary Files

Supplementary data 1

Supplementary data 2

Supplementary data 3

Supplementary data 4

Supplementary data 5

Reporting summary

## Data Availability

All sequencing data is freely available at the European Nucleotide Archive (https://www.ebi.ac.uk/ena/browser/home) under the sequencing project IDs ERP120687 (short read data; accessions in Supplementary Data 1) and PRJNA1121524 (long-read data; accessions in Supplementary Data 2), detailed per-isolate information is provided in Supplementary Data 3. Blood culture and CSF data used to show the trends and numbers of *E. coli* cases per year is available in Supplementary Data 4. Antimicrobial resistance gene data is available in Supplementary Data 5. Supplementary Data and codes for reproducing the figures are also available at GitHub (https://github.com/ohapearse/invasive_neonatal_ecoli; 10.5281/zenodo.15622585). All other information is available from the corresponding authors upon reasonable request.

## Code availability

Codes for reproducing the figures are available at GitHub (https://github.com/ohapearse/invasive_neonatal_ecoli; 10.5281/zenodo.15622585).
