# Supplementary material for: High diversity of Escherichia coli causing invasive disease in neonates in Malawi poses challenges for O-antigen based vaccine approach
Source: Commun Med (Lond). 2025 Jul 18;5:298. doi: 10.1038/s43856-025-01007-1 (PMC12274568; doi:10.1038/s43856-025-01007-1)
Supplement: Supplementary file 1 — Supplementary information [file 43856_2025_1007_MOESM1_ESM.docx]

**Supplementary information**

High diversity of *Escherichia coli* causing invasive disease in neonates in Malawi poses challenges for O-antigen based vaccine approach

Oliver Pearse^1,2^, Allan Zuza^2^, Edith Tewesa^3^, Patricia Siyabu^3^, Alice J Fraser^4^, Jennifer Cornick^2,5^, Kondwani Kawaza^2,6^, Patrick Musicha^2,4,7^, Nicholas R Thomson^7,8^, Nicholas A Feasey^1,2,9^*, Eva Heinz^1,4,10^*

*Equally contributing

### Affiliations:

1. Department of Clinical Sciences, Liverpool School of Tropical Medicine, Liverpool, UK

2. Malawi-Liverpool-Wellcome Programme, Kamuzu University of Health Sciences, Blantyre, Malawi

3. Queen Elizabeth Central Hospital, Blantyre, Malawi

4. Department of Vector Biology, Liverpool School of Tropical Medicine, Liverpool, UK

5. University of Liverpool, Institute of Infection, Veterinary and Ecological Sciences, Liverpool, UK

6. Kamuzu University of Health Sciences, Blantyre, Malawi

7. Wellcome Sanger Institute, Parasites and Microbes Program, Hinxton, UK

8. London School of Tropical Medicine and Hygiene, Department of Pathogen Molecular Biology, London, UK

9. The School of Medicine, University of St. Andrews, St. Andrews, UK

10. University of Strathclyde, Strathclyde Institute for Pharmacy and Biomedical Sciences, Glasgow, UK

### **Corresponding author email:** [oliver.pearse@lstmed.ac.uk](mailto:oliver.pearse@lstmed.ac.uk); [eva.heinz@strath.ac.uk](mailto:eva.heinz@strath.ac.uk)

**Supplementary methods**

**Details on microbiological sample processing**

Briefly, 1-2mL of blood was taken from neonates (up to 28 days old) with risk factors for sepsis (i.e. maternal fever during labour, prolonged rupture of membranes, tachypnoea), or clinical suspicion of sepsis (fever >38^∘^C, tachypnoea, tachycardia, reduced activity, seizures). For some clinical records, information on age was only described in months of age and individuals whose age was entered as being ‘1 month’ were also considered as neonates for the purposes of our study. For neonates with clinical suspicion of sepsis or other clinical suspicion of meningitis (raised fontanelle, abnormal neurology), a lumbar puncture was also performed. Five pairs of samples may have been from the same patient, although this is only an estimate based on comparing ST, ward and date of samples taken, as no patient IDs exist in Malawi and each sample is stored under a separate, novel laboratory ID not linked to the patient identity other than by name. Blood was collected using aseptic methods and inoculated into a single aerobic bottle (BacT/Alert, bioMérieux, Marcy-L’Etoile, France), then incubated using the automated BacT/Alert system. Samples that flagged positive were Gram stained and Gram-negative bacilli were identified by Analytical Profile Index (bioMérieux). Occasionally more than one *E. coli* colony morphology was identified from a sample, in which case both were stored. Of 207 *E. coli* isolates in the study period, 192 could be successfully regrown and were recovered for WGS, and 170 passed QC. One sequenced isolate was a duplicate, leaving 169 isolates for further analysis.

**DNA preparation for sequencing**

Single-morphology plates had a single colony pick taken and inoculated into 15ml of buffered peptone water for 18-24 hours at 37°C. These samples were then centrifuged and the supernatant was discarded. The pellet was then resuspended in buffer. For the short-read sequencing the DNA was extracted using the QIAsymphony machine and QIAsymphony DSP kit with onboard lysis, according to the manufacturer’s instructions. Quality control was done using Qubit and samples with a DNA volume of less than 200ng were repeated. Samples that passed QC underwent Whole Genome Sequencing (WGS) at the Wellcome Sanger Institute. For long-read sequencing of selected isolates, DNA was extracted using the MasterPure Complete DNA and RNA isolation kit following the manufacturer’s instructions for the purification of DNA from cell samples. DNA was then quality controlled using the Qubit dsDNA Broad Range assay and the TapeStation (4150) system, using the Genomic DNA Screen Tape Kit.

**Genome sequence QC and assembly**

Species confirmation was performed using Kraken v1.1.120^1^, and any sample with greater than 5% read content other than *E. coli* or Unclassified was excluded. Annotated assemblies for the short read data were produced using the pipeline described previously^2^. De novo assembly of genome sequences was performed using SPAdes v3.14.0^3^, trialing different kmer lengths between 41 and 127 to find the optimal kmer length. An assembly improvement step was applied to the assembly with the best N50 and contigs scaffolded using SSPACE v2.023^4^ and sequence gaps filled using GapFiller v1.1124^5^. Assembly statistics were generated using the Sanger Pathogens pipeline as available on github^6^ (https://github.com/sanger-pathogens/assembly-stats).Samples with <20 or >200 contigs and a genome size of <4.4MB or >5.6MB were excluded. Isolates with greater than 5% heterozygous SNPs of the total genome were also excluded due to potential within-species contamination. Automated annotation was performed using PROKKA v1.5^7^ and genus specific databases from RefSeq^8^. The improved assembly step uses software developed by the Pathogen Informatics team at the WSI which is freely available for download from github (https://github.com/sanger-pathogens) under an open-source license, GNU GPL 3. The improvement step of the pipeline is also available as a standalone Perl module from CPAN (<https://metacpan.org/author/AJPAGE>). For long-read sequence data, basecalling and demultiplexing on raw long-reads was performed with guppy v2.6.1 (https://nanoporetech.com/software/other/guppy) using the super-accurate model for basecalling, adapters removed with porechop v 0.2.4^9^, and low-quality reads were removed with filtlong v0.2.238^10^ (https://github.com/rrwick/Filtlong) before assembly. Long-read-first hybrid assemblies from isolates sequenced on the Oxford Nanopore platform were produced using Flye v 2.9.339^11^, then visualised with Bandage v0.8.1^12^. Long-read polishing was performed using Medaka v1.8.0^13^ (https://github.com/nanoporetech/medaka), then short-read polished with Polypolish v0.6.0^14^ and Pypolca v0.3.0^15^. Assembled genomes were annotated using prokka as described above.

17 isolates were excluded due to contamination, and 17 were excluded due to failure of assembly or poor assembly metrics (twelve isolates failed both, and five of each just failed due to one reason). There were no isolates that were excluded due to potential within-species contamination. There was one duplicate isolate (two sequencing runs of the same isolate from the same sample), of which one isolate was removed from analysis, so the total number of isolates analysed was 169.

**Details on resistance mechanisms of drugs not routinely used in neonates**

There were several isolates with fluoroquinolone resistance mutations, the most frequent were the *gyrA* mutations *gyrA_*S38L found in 38/169 (22.5%) isolates and *gyrA_*D87N found in 24/169 (14.2%) isolates, the *parC* mutation *parC*_S80I found in 26/169 (15.4%) (Supplementary Figure 3). We further note 37/169 (21.9%) isolates with *parE* mutations which are not described as sufficient to provide resistance for *E. coli*, but could lead to reduced susceptibility or higher resistance levels if an additional mutation is present. All ST410 isolates encoded four different, acquired fluoroquinolone resistance genes (two *gyrA* mutations, one *parC* and one *parE* each). Likewise, we identified at least one acquired fluoroquinolone resistance gene in all ST131 isolates *gyrA*, *parC* and *parE*). Fluoroquinolone resistance genes (*gyrA*, *parC* and *qnrS1*) were found in 8/20 (40%) of ST69 isolates and 3/15 (20%) of ST10 isolates. The most frequently occurring chloramphenicol genes were *cat*A1 found in 22/169 (13.0%) isolates and *cat*B3, found in 20/169 (11.8%) isolates (Supplementary Figure 3). Colistin resistance was not tested in our cohort, but genes conferring colistin resistance were found relatively frequently, with *pmr*B_E123D found in 48/169 (28.4%) isolates and *pmr*B_Y358N found in 36/169 (21.3%) isolates (Supplementary Figure 3).

## **Supplementary figures**


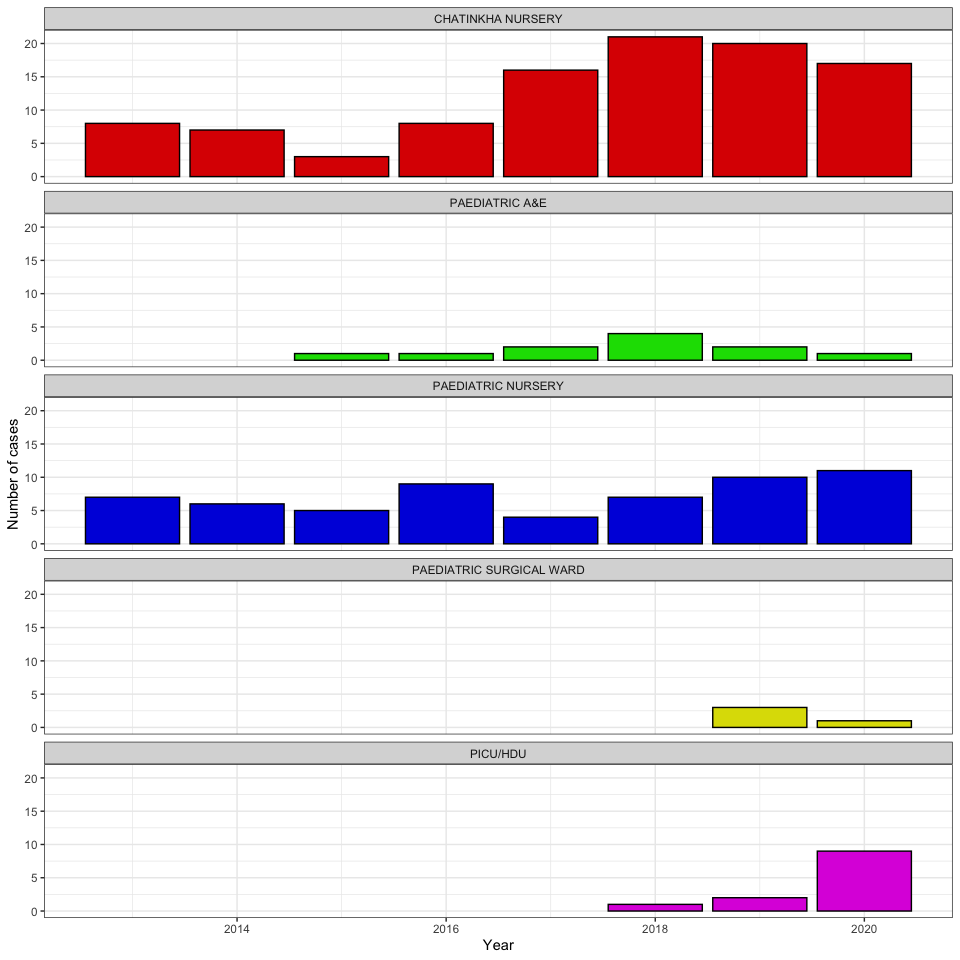


**Supplementary Figure 1. E. coli cases per year by ward.**

The number of cases (y-axis) stratified by ward; Chatinka Nursery (red; n=112), Paediatric Accident and Emergency (A&E) (green; n=12), Paediatric Nursery (blue; n=62), Paediatric Surgical Ward (yellow; n=4) and Paediatric Intenstive Care Unit/High Dependency Unit (PICU/HDU) (pink; n=12) over time (x-axis).


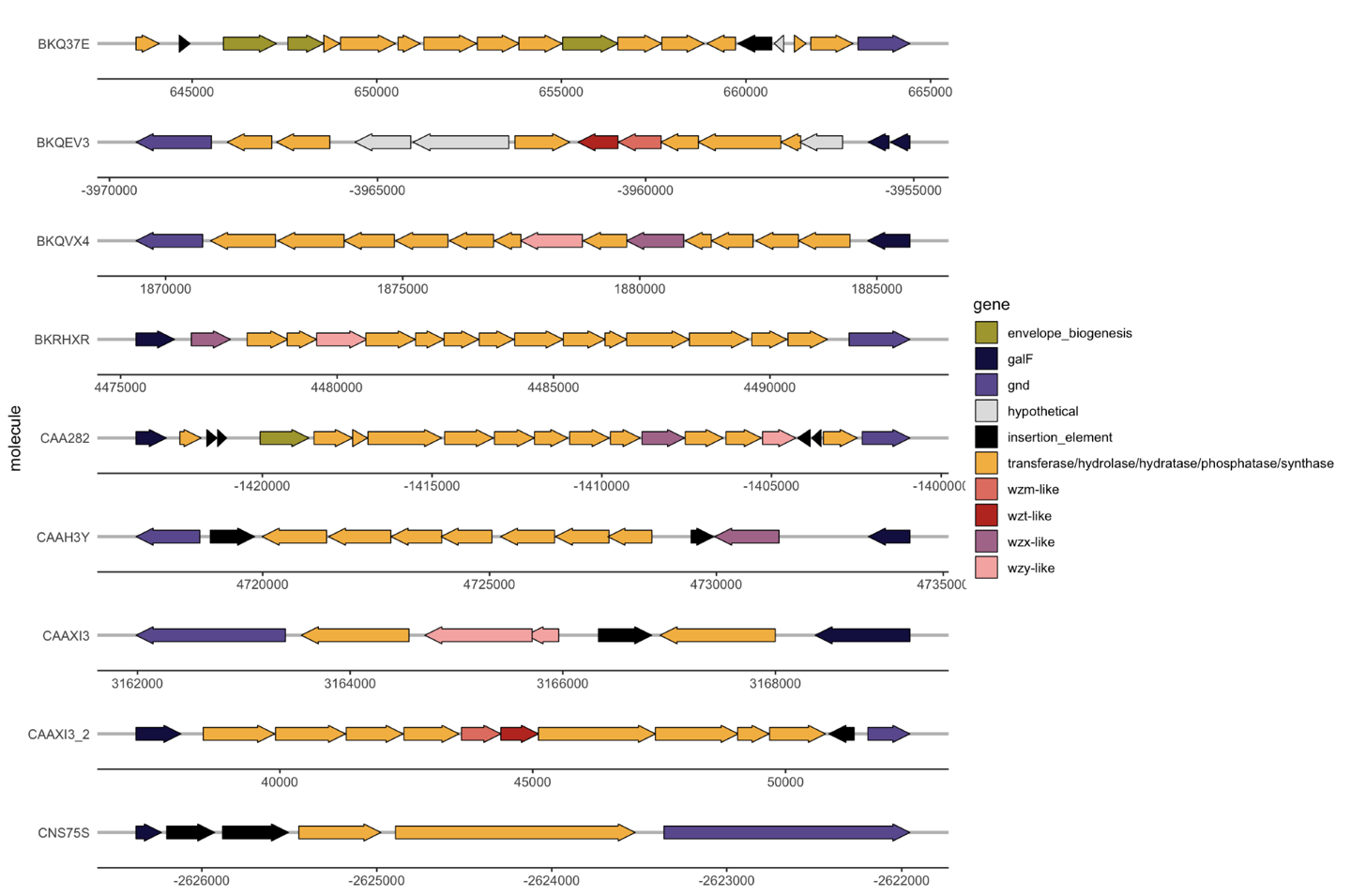


**Supplementary Figure 2. A schematic showing the operon structure of the novel O-antigen genes.**

Each row represents a different O-type not found in the databases used for O-type identification from a single isolate. For isolates with > 0.9 sequence homology for the O-antigen gene, only one isolate was selected for representation. Three of the isolates (CAAH3Y, CNS75S and BKQ37E) have loci heavily disrupted by insertion elements and seem to lack the components for an export machinery (wzm/wzt or wzx/wzy). It remains to be investigated whether they acquired an entirely unrelated O-antigen locus from a different organism that integrated into a different part of the genome and encodes for export machineries sufficiently different to not be recognized by read-based searches, or whether these isolates indeed do not encode for a classical O-antigen. One of the isolates (CAAXI3) has a similarly disrupted galF/gnd site but encodes for a potentially functional O-antigen locus on a plasmid (CAAXI3_2), raising interesting questions regarding the expression of this O-antigen locus and whether this will remain plasmid-located or eventually become integrated into the disrupted chromosomal location.


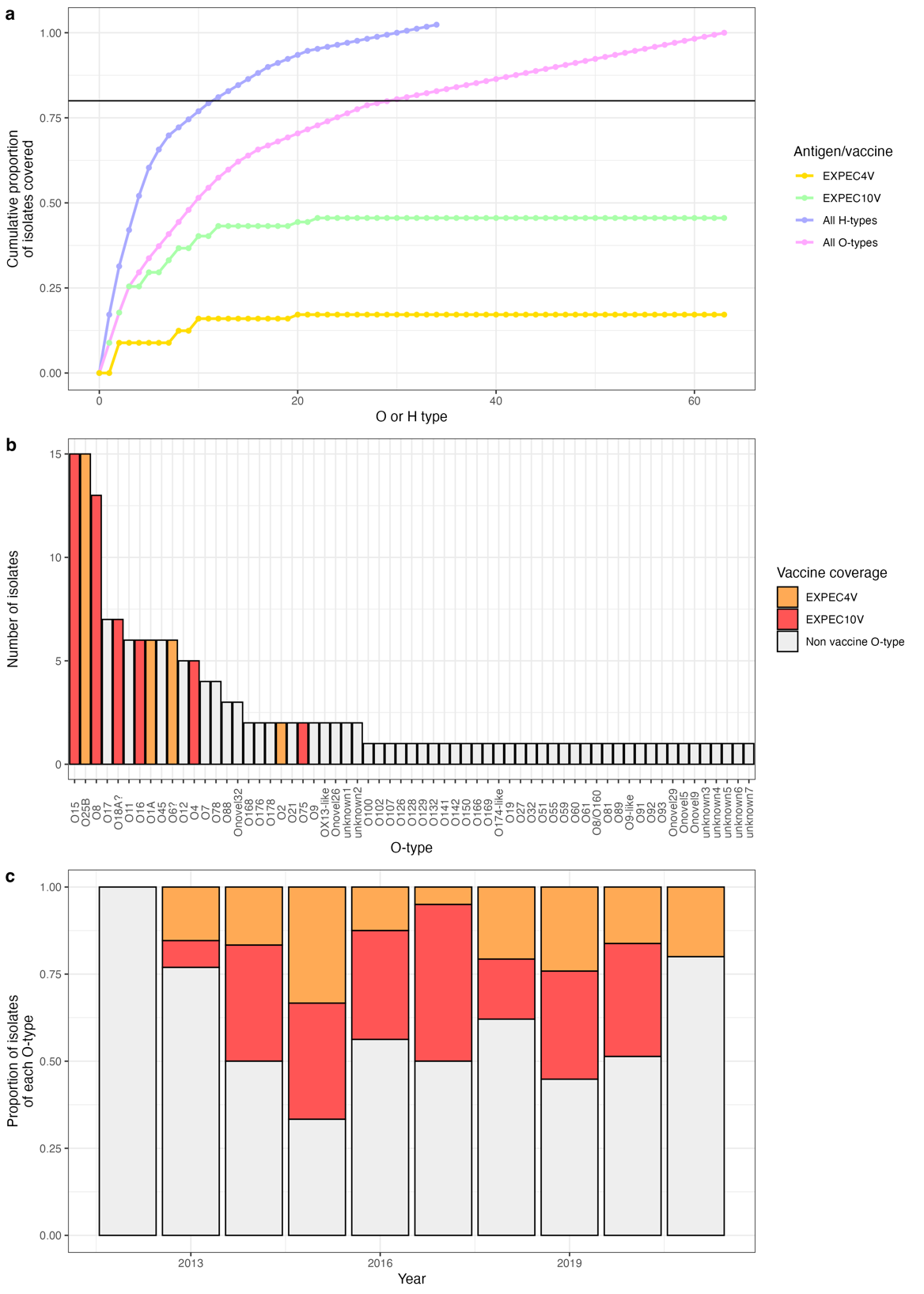


**Supplementary Figure 3. Theoretical vaccine coverage for Extra-intestinal Pathogenic E. coli 10-valent (EXPEC10V) and EXPEC4V**

**A)** Rarefaction curve showing the theoretical protection given against vaccines covering the most frequently isolated H-types and O-types, as well as the potential protection given by the EXPEC10V and EXPEC4V. The horizontal line shows the point at which 80% of isolates would be covered. For isolates with more than one H-type both were counted, there were multiple isolates with more than one H-type so the line for H-type goes above 1. **B)** A bar chart showing the frequency of the different O-types. **C)** A bar chart showing the proportion of isolates per year that had different O-types. The colours are the same as those represented in Figure 4B.


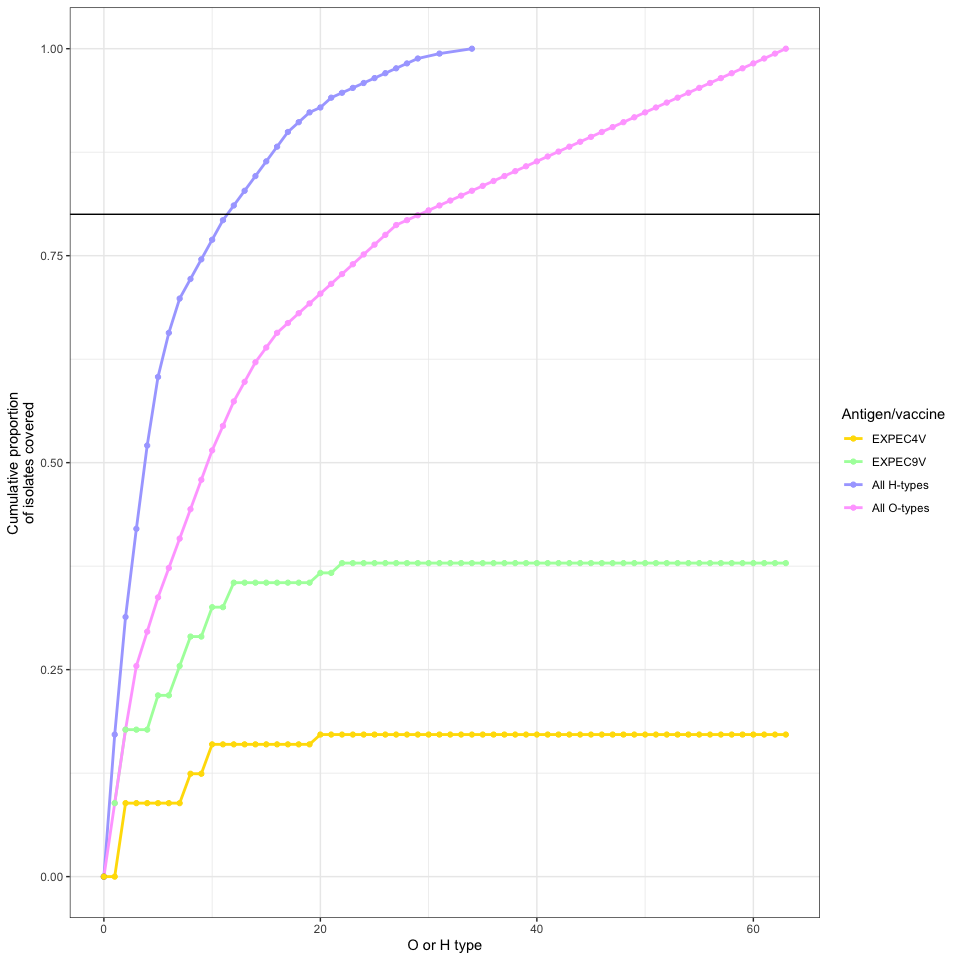


**Supplementary Figure 4. Theoretical vaccine coverage for Extra-intestinal Pathogenic E. coli 9-valent (EXPEC9V) and EXPEC4V, alternative analysis**

Rarefaction curve showing the theoretical protection given against vaccines covering the most frequently isolated H-types and O-types, as well as the potential protection given by the EXPEC9V and EXPEC4V. The horizontal line shows the point at which 80% of isolates would be covered. For isolates with more than one H-type only the most frequently occurring H-type was counted.


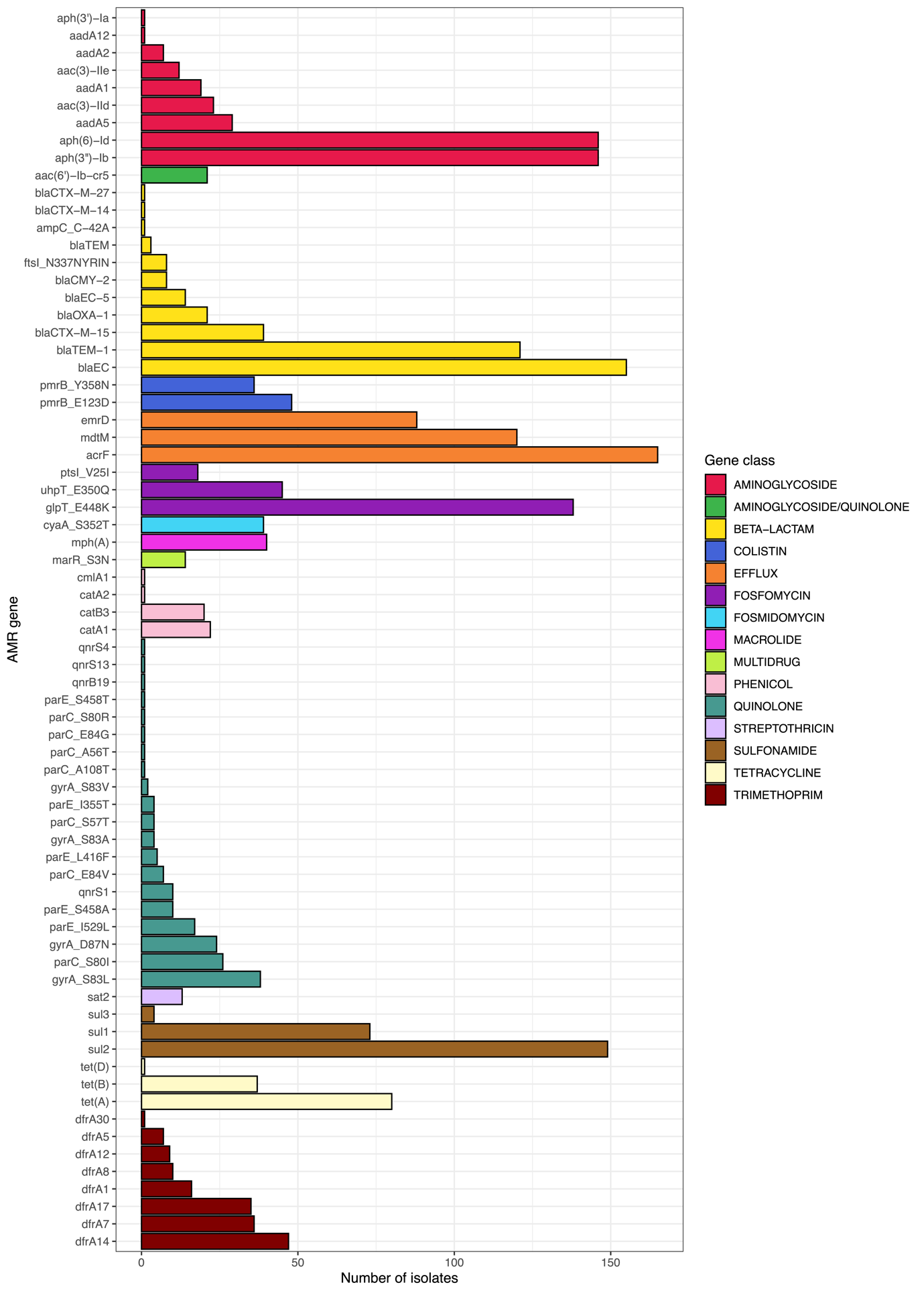


**Supplementary Figure 5. Frequency of antimicrobial resistance (AMR) genes in the collection separated by class.**

***Supplementary Table 1. A table showing the AMR genes present in study isolates for different AMR classes.***

The first column describes the AMR gene classes and the second column describes the AMR genes. Gene names have been italicized.

| AMR class | AMR genes |
| --- | --- |
| Amikacin | *aac(6')-Ib-cr5* |
| Aminoglycoside | *aph(3'')-Ib, aph(6)-Id, aadA5, aac(3)-IId, aadA1, aac(3)-IIe, aadA2, aadA12, aph(3')-Ia* |
| Beta-Lactam | *bla_EC_, bla_TEM-1_, bla_OXA-1_, bla_EC-5_, bla_CMY-2_, ftsI_N337NYRIN, ampC_C-42A* |
| Colistin | *pmrB_E123D, pmrB_Y358N* |
| Extended-spectrum Beta-Lactam | *bla_CTX-M-15_, bla_CTX-M-14_, bla_CTX-M-27_* |
| Fosfomycin | *glpT_E448K, uhpT_E350Q, ptsI_V25I* |
| Phenicol | *catA1, catB3, catA2, cmlA1* |
| Quinolone | *gyrA_S83L, parC_S80I, gyrA_D87N, parE_I529L, parE_S458A, qnrS1, parC_E84V, parE_L416F, gyrA_S83A, parC_S57T, parE_I355T, gyrA_S83V, parC_A108T, parC_A56T, parC_E84G, parC_S80R, parE_S458T, qnrB19, qnrS13, qnrS4* |
| Sulfonamide | *sul2, sul1, sul3* |
| Tetracycline | *tet(A), tet(B), tet(D)* |
| Trimethoprim | *dfrA14, dfrA7, dfrA17, dfrA1, dfrA8, dfrA12, dfrA5, dfrA30* |

**Supplementary References**

1. Wood, D. E. & Salzberg, S. L. Kraken: ultrafast metagenomic sequence classification using exact alignments. *Genome Biol.* **15**, R46 (2014).

2. Page, A. J. *et al.* Robust high-throughput prokaryote de novo assembly and improvement pipeline for Illumina data. *Microb. Genomics* **2**, e000083 (2016).

3. Bankevich, A. *et al.* SPAdes: a new genome assembly algorithm and its applications to single-cell sequencing. *J. Comput. Biol. J. Comput. Mol. Cell Biol.* **19**, 455–477 (2012).

4. Boetzer, M., Henkel, C. V., Jansen, H. J., Butler, D. & Pirovano, W. Scaffolding pre-assembled contigs using SSPACE. *Bioinforma. Oxf. Engl.* **27**, 578–579 (2011).

5. Boetzer, M. & Pirovano, W. Toward almost closed genomes with GapFiller. *Genome Biol.* **13**, R56 (2012).

6. Pathogen Informatics, Wellcome Sanger Institute. assembly-stats.

7. Seemann, T. Prokka: rapid prokaryotic genome annotation. *Bioinforma. Oxf. Engl.* **30**, 2068–2069 (2014).

8. Pruitt, K. D., Tatusova, T., Brown, G. R. & Maglott, D. R. NCBI Reference Sequences (RefSeq): current status, new features and genome annotation policy. *Nucleic Acids Res.* **40**, D130–D135 (2012).

9. Wick, R. R., Judd, L. M., Gorrie, C. L. & Holt, K. E. Completing bacterial genome assemblies with multiplex MinION sequencing. *Microb. Genomics* **3**, (2017).

10. Wick, R. R. Filtlong.

11. Kolmogorov, M., Yuan, J., Lin, Y. & Pevzner, P. A. Assembly of long, error-prone reads using repeat graphs. *Nat. Biotechnol.* **37**, 540–546 (2019).

12. Wick, R. R., Schultz, M. B., Zobel, J. & Holt, K. E. Bandage: interactive visualization of *de novo* genome assemblies. *Bioinformatics* **31**, 3350–3352 (2015).

13. nanoporetech. medaka. (2018).

14. Wick, R. R. & Holt, K. E. Polypolish: Short-read polishing of long-read bacterial genome assemblies. *PLOS Comput. Biol.* **18**, e1009802 (2022).

15. Zimin, A. V. & Salzberg, S. L. The genome polishing tool POLCA makes fast and accurate corrections in genome assemblies. *PLOS Comput. Biol.* **16**, e1007981 (2020).
