# Supplementary material for: High diversity of Escherichia coli causing invasive disease in neonates in Malawi poses challenges for O-antigen based vaccine approach
Source: Commun Med (Lond). 2025 Jul 18;5:298. doi: 10.1038/s43856-025-01007-1 (PMC12274568; doi:10.1038/s43856-025-01007-1)
Supplement: Supplementary file 2 — Description of Additional Supplementary Files [file 43856_2025_1007_MOESM2_ESM.pdf]

Description of Additional Supplementary Files

File name: Supplementary Data 1

Description: Data under sequencing project ID ERP120687 (short read data)

File name: Supplementary Data 2

Description: Data under sequencing project ID PRJNA1121524 (long-read data)

File name: Supplementary Data 3

Description: detailed per-isolate information

File name: Supplementary Data 4

Description: Blood culture and CSF data used to show the trends and numbers of *E. coli* cases per year

File name: Supplementary Data 5

Description: Antimicrobial resistance gene data
