# Supplementary material for: High diversity of Escherichia coli causing invasive disease in neonates in Malawi poses challenges for O-antigen based vaccine approach
Source: Commun Med (Lond). 2025 Jul 18;5:298. doi: 10.1038/s43856-025-01007-1 (PMC12274568; doi:10.1038/s43856-025-01007-1)
Supplement: Supplementary file 8 — Reporting summary [file 43856_2025_1007_MOESM8_ESM.pdf]

nature portfolio

Corresponding author(s): Eva Heinz

Last updated by author(s): Jun 9, 2025

Reporting Summary

Nature Portfolio wishes to improve the reproducibility of the work that we publish. This form provides structure for consistency and transparency in reporting. For further information on Nature Portfolio policies, see our Editorial Policies and the Editorial Policy Checklist.

Statistics

For all statistical analyses, confirm that the following items are present in the figure legend, table legend, main text, or Methods section.

n/a	Confirmed	
☐	☒	The exact sample size (*n*) for each experimental group/condition, given as a discrete number and unit of measurement
☐	☒	A statement on whether measurements were taken from distinct samples or whether the same sample was measured repeatedly
☐	☒	The statistical test(s) used AND whether they are one- or two-sided<br>*Only common tests should be described solely by name; describe more complex techniques in the Methods section.*
☐	☒	A description of all covariates tested
☐	☒	A description of any assumptions or corrections, such as tests of normality and adjustment for multiple comparisons
☐	☒	A full description of the statistical parameters including central tendency (e.g. means) or other basic estimates (e.g. regression coefficient) AND variation (e.g. standard deviation) or associated estimates of uncertainty (e.g. confidence intervals)
☐	☒	For null hypothesis testing, the test statistic (e.g. *F*, *t*, *r*) with confidence intervals, effect sizes, degrees of freedom and *P* value noted<br>*Give P values as exact values whenever suitable.*
☐	☒	For Bayesian analysis, information on the choice of priors and Markov chain Monte Carlo settings
☐	☒	For hierarchical and complex designs, identification of the appropriate level for tests and full reporting of outcomes
☐	☒	Estimates of effect sizes (e.g. Cohen's *d*, Pearson's *r*), indicating how they were calculated

Our web collection on statistics for biologists contains articles on many of the points above.

Software and code

Policy information about availability of computer code

Data collection	The methods section provides details on all software and version used.
Data analysis	The methods section provides details on all software used and any relevant settings. Supplementary Data and codes for reproducing the figures is also available at github (https://github.com/ohapearse/invasive_neonatal_ecoli; DOI: https://doi.org/10.5281/zenodo.15622585).

For manuscripts utilizing custom algorithms or software that are central to the research but not yet described in published literature, software must be made available to editors and reviewers. We strongly encourage code deposition in a community repository (e.g. GitHub). See the Nature Portfolio guidelines for submitting code & software for further information.

Data

Policy information about availability of data

All manuscripts must include a data availability statement. This statement should provide the following information, where applicable:

- Accession codes, unique identifiers, or web links for publicly available datasets
- A description of any restrictions on data availability
- For clinical datasets or third party data, please ensure that the statement adheres to our policy

All sequencing data is freely available under the sequencing project IDs ERP120687 (short read data; accessions in Supplementary Table 1) and PRJNA1121524 (long-read data; accessions in Supplementary Table 2), detailed per-isolate information is provided in Supplementary Table 3. Blood culture and CSF data used to

show the trends and numbers of E. coli cases per year is available in Supplementary Table 4. Supplementary Data and codes for reproducing the figures is also available at github (https://github.com/ohapearse/invasive_neonatal_ecoli; DOI: https://doi.org/10.5281/zenodo.15622585).

Human research participants

Policy information about studies involving human research participants and Sex and Gender in Research.

Reporting on sex and gender	We identified 201 E. coli isolated from neonates in the period from September 2012 to March 2021 (Figure 2C); 95/201 (47.3%) were female, with a median age of 3 [IQR 2 - 8] days (Figure 1D).
Population characteristics	Routine, quality assured diagnostic blood culture services have been provided to the medical and paediatric wards by the Malawi-Liverpool-Wellcome Programme (MLW) since 1998. Briefly, 1-2mL of blood was taken from neonates (up to 28 days old) with risk factors for sepsis (i.e. maternal fever during labour, prolonged rupture of membranes, tachypnoea), or clinical suspicion of sepsis (fever >38^?C, tachypnoea, tachycardia, reduced activity, seizures). For some clinical records, information on age was only described in months of age and individuals whose age was entered as being '1 month' were also considered as neonates for the purposes of our study.
Recruitment	These were stored bacterial isolates from routine diagnostics.
Ethics oversight	This study was ethically approved by the Kamuzu University of Health Sciences College of Medicine Research Ethics Committee (COMREC P.06.20.3071).

Note that full information on the approval of the study protocol must also be provided in the manuscript.

Field-specific reporting

Please select the one below that is the best fit for your research. If you are not sure, read the appropriate sections before making your selection.

☒ Life sciences ☐ Behavioural & social sciences ☐ Ecological, evolutionary & environmental sciences

For a reference copy of the document with all sections, see nature.com/documents/nr-reporting-summary-flat.pdf

Life sciences study design

All studies must disclose on these points even when the disclosure is negative.

Sample size	The MLW database was screened from 2000 to 2021 to identify all cases of E. coli infection in the hospital, and all E. coli isolates from neonates (recorded as less than 29 days old or as 1 month old on ledgers) in the period from September 2012 to March 2021 were selected for whole genome sequencing. This time period was chosen as this was the time period for which we had consistent metadata at the time of whole genome sequencing.
Data exclusions	No growth of the isolates, quality failure during DNA extraction or sequencing
Replication	Not applicable for this study (surveillance of bacterial infections)
Randomization	Not applicable for this study (surveillance of bacterial infections)
Blinding	Not applicable for this study (surveillance of bacterial infections)

Reporting for specific materials, systems and methods

We require information from authors about some types of materials, experimental systems and methods used in many studies. Here, indicate whether each material, system or method listed is relevant to your study. If you are not sure if a list item applies to your research, read the appropriate section before selecting a response.

Materials & experimental systems

n/a	Involved in the study
☒	☐ Antibodies
☒	☐ Eukaryotic cell lines
☒	☐ Palaeontology and archaeology
☒	☐ Animals and other organisms
☒	☐ Clinical data
☒	☐ Dual use research of concern

Methods

n/a	Involved in the study
☒	☐ ChIP-seq
☒	☐ Flow cytometry
☒	☐ MRI-based neuroimaging
